# Supplementary material for: Left Heart Disease Phenotype in Elderly Patients with Pulmonary Arterial Hypertension: Insights from the Italian PATRIARCA Registry
Source: J Clin Med. 2022 Nov 30;11(23):7136. doi: 10.3390/jcm11237136 (PMC9735657; doi:10.3390/jcm11237136)
Supplement: Supplementary file 1 [file jcm-11-07136-s001.zip › jcm-2032160-supplementary.pdf]

Left heart disease phenotype in elderly patients with pulmonary arterial hypertension: insights from the Italian PATRIARCA registry.

Matteo Toma, Roberta Miceli, Edoardo Bonsante, Davide Colombo, Marco Confalonieri, Andrea Garascia, Stefano Ghio, Mariangela Lattanzio, Carlo Maria Lombardi, Giuseppe Paciocco, Cristina Piccinino, Irene Rota, Caterina Santolamazza, Laura Scelsi, Piermario Scuri, Davide Stolfo, Antonella Vincenzi, Lorenzo Volpiano, Marco Vicenzi and Pietro Ameri.

SUPPLEMENTARY MATERIALS

Table of contents

Table S1: Patient enrollment across the PATRIARCA registry.

Table S2: Baseline characteristics according to the presence of the main analysis clinical criteria suggestive for left heart disease.

Table S3. Baseline characteristics according to the presence of the main analysis hemodynamic criteria suggestive for left heart disease.

Table S4. Baseline characteristics of the study population according to the secondary analysis.

Table S5. Baseline characteristics of the study population according to the secondary analysis clinical criteria suggestive for left heart disease.

Table S1. Patient enrolment across the PATRIARCA registry.

Center	Enrolled patients (N=180)	Included in the analysis (N=69)
Cardiology, IRCCS Policlinico San Matteo, Pavia	56 (31)	23 (33)
Cardiology, IRCCS Policlinico San Martino, Genova	28 (16)	9 (13)
Cardiology, Ospedale San Gerardo, Monza	20 (11)	9 (13)
Cardiology, Ospedale di Bolzano	14 (8)	2 (3)
Cardiology, IRCCS Ca' Granda Ospedale Maggiore Policlinico, Milano	12 (7)	3 (4)
ASST Grande Ospedale Metropolitano Niguarda, Milano	12 (7)	8 (12)
Cardiology, Azienda sanitaria Universitaria Giuliano Isontina, Trieste	10 (6)	4 (6)
Cardiology, ASST degli Spedali Civili, Brescia	10 (6)	2 (3)
Cardiology, Ospedale di Circolo e Fondazione Macchi, Varese	9 (5)	4 (6)
Cardiology, ASST Papa Giovanni XXIII, Bergamo	5 (3)	3 (4)
Cardiology, Ospedale Maggiore della Carità, Novara	4 (2)	2 (3)

Patient enrollment across the 11 centers participating in the Italian multicenter PATRIARCA registry between December 1st 2019 and September 15th 2020.

Data are expressed as count (%).

Table S2. Baseline characteristics according to the presence of the main analysis clinical criteria suggestive for left heart disease.

	**Overall** (N=69)	**No clinical LHD profile** (N=63)	**Clinical LHD phenotype** (N=6)	**P**
Demographics				
Age at diagnosis, y	73 ± 4	73 ± 4	74 ± 3	0.58
Age at enrolment, years	77 ± 5	77 ± 4	77 ± 3	0.87
Female	44 (64)	42 (67)	2 (33)	0.12
Weight, Kg	64 ± 15	64 ± 15	70 ± 12	0.34
Height, cm	163 ± 9	162 ± 8	169 ± 11	0.07
BSA, m^2	1.70 ± 0.22	1.69 ± 0.22	1.81 ± 0.18	0.21
BMI, Kg/m^2	24 ± 5	24 ± 5	25 ± 4	0.82
Clinical and echocardiographic parameters				
WHO-FC I-II	32 (46)	30 (48)	2 (33)	0.38
Systemic hypertension	45 (65)	39 (62)	6 (100)	0.07
Diabetes	15 (22)	9 (14)	6 (100)	**<0.001**
CAD	17 (25)	12 (19)	5 (83)	**0.003**
Permanent AF	3 (4)	3 (5)	6 (100)	0.76
Pulmonary disease	31 (45)	27 (43)	4 (67)	0.25

CKD	26 (38)	21 (33)	5 (83)	**0.03**
Previous/current cancer	8 (12)	8 (13)	6 (100)	0.46
SBP, mmHg	124 ± 15	124 ± 14	122 ± 19	0.71
DBP, mmHg	72 ± 9	72 ± 9	67 ± 10	0.19
SO_2, %	95 [93; 97]	95 [93; 97]	96 [88; 97]	1
6MWD, meters	304 ± 199	305 ± 123	290 ± 73	0.81
LVEF <50%	4 (6)	3 (5)	1 (17)	0.25
LVH	17 (25)	16 (25)	1 (17)	0.53
LA dilation	31 (45)	28 (44)	3 (50)	0.59
TAPSE, mm	20 ± 5	21 ± 4	17 ± 5	**0.03**
TRV, m/s	3.81 ± 0.76	3.78 ± 0.76	4.10 ± 0.74	0.32
TAPSE/TRV	5.6 ± 1.9	5.8 ± 1.9	4.1 ± 1.2	0.05
RVSP, mmHg	57 [42; 77]	57 [40; 75]	73 [45; 90]	0.29
RA dilation	53 (77)	47 (75)	6 (100)	0.25
Pericardial effusion	11 (16)	9 (14)	2 (33)	0.24
Most recent RHC				
RAP, mmHg	7 [4; 10]	7 [4; 10]	6 [2; 10]	0.58
mPAP, mmHg	41 ± 10	41 ± 11	41 ± 10	0.98
dPAP, mmHg	25 ± 9	26 ± 9	23 ± 6	0.58
sPAP, mmHg	70 ± 20	69 ± 20	74 ± 20	0.53
PAWP, mmHg	11 ± 4	12 ± 4	7 ± 2	**0.004**
RAP/PAWP ratio	0.60 [0.46; 0.75]	0.58 [0.41; 0.75]	0.73 [0.67; 1.00]	0.18
PVR, WU	7.39 ± 4.53	7.22 ± 4.54	9.14 ± 4.47	0.33
Cardiac output, L/min	4.59 ± 1.43	4.65 ± 1.46	4.04 ± 1.04	0.33
Cardiac index, L/min/m^2	2.74 ± 0.81	2.78 ± 0.82	2.30 ± 0.62	0.17
Diagnosis to last RHC interval, months	15 [4; 33]	15 [4; 35]	13 [0; 23]	0.42
Treatment				
No PAH therapy	6 (9)	5 (8)	1 (17)	0.43
Bosentan	-	-	-	
Ambrisentan	17 (25)	16 (25)	1 (17)	0.54
Macitentan	32 (46)	29 (46)	3 (50)	0.61
ERA	49 (71)	45 (71)	4 (67)	0.57
Sildenafil	22 (32)	19 (30)	3 (50)	0.29
Tadalafil	25 (36)	24 (38)	1 (17)	0.29
Riociguat	3 (4)	3 (5)	0	0.76
PDE5i/GCs	50 (73)	46 (73)	4 (67)	0.53
Dual oral combination therapy	36 (52)	33 (52)	3 (50)	0.62
Selexipag	6 (9)	6 (10)	0	0.57
Treprostinil	-	-	-	
Epoprostenol i.v.	1 (1)	1 (2)	0	0.91

Inhaled iloprost	2 (3)	2 (3)	0	0.83
Beta blockers	9 (13)	7 (11)	2 (33)	0.17
RASi	23 (33)	21 (33)	2 (33)	0.69
MRA	32 (46)	26 (41)	6 (100)	**0.007**
Furosemide	57 (83)	51 (81)	6 (100)	0.24
Digoxin	6 (9)	6 (10)	0	0.57
Amiodarone	9 (13)	9 (14)	0	0.41
Warfarin	11 (16)	10 (16)	1 (17)	0.67
DOAC	11 (16)	10 (16)	1 (17)	0.67
SAPT	22 (32)	18 (29)	4 (67)	0.07
Statins	26 (38)	22 (35)	4 (67)	0.14
Ezetimibe	3 (4)	1 (2)	2 (33)	**0.02**
Antidiabetic drugs	12 (17)	6 (10)	6 (100)	**<0.001**

Characteristics of patients with and without a left heart disease (LHD) phenotype according to the clinical criteria definition in the main analysis. Data are expressed as n (%), mean ± SD or median [IQR], as appropriate.

LHD, left heart disease; *BSA,* body surface area; *BMI,* body mass index; *PAH,* pulmonary arterial hypertension; *WHO-FC,* World Health Organization functional class; *CAD,* coronary artery disease; *AF,* atrial fibrillation; *CKD,* chronic kidney disease; *SBP,* systolic blood pressure; *DBP,* diastolic blood pressure; *SO_2,* oxygen saturation; *6MWD,* six minute walking distance; *LVEF*, left ventricular ejection fraction; *LVH*, left ventricular hypertrophy; *LA,* left atrium; *TAPSE,* tricuspid annular plane systolic excursion; *TRV,* tricuspid regurgitant velocity; *RVSP,* right ventricular systolic pressure; *RA,* right atrium; *RHC,* right heart catheterization; *RAP,* right atrial pressure; *mPAP, dPAP and sPAP* for mean, diastolic and systolic pulmonary artery pressure; *PAWP,* pulmonary artery wedge pressure; *PVR,* pulmonary vascular resistance; *ERA,* endothelin receptor antagonist; *PDE5i,* phosphodiesterase type 5 inhibitor; *GCs,* guanylate cyclase stimulator; *RASi,* renin-angiotensin system inhibitors; *MRA,* mineralcorticoid receptor antagonist; *DOAC,* direct oral anticoagulant; *SAPT,* single antiplatelet therapy.

Table S3. Baseline characteristics according to the presence of the main analysis hemodynamic criteria suggestive for left heart disease.

	**Overall (N=69)**	**No hemodynamic LHD profile (N=52)**	**Hemodynamic LHD phenotype (N=17)**	**P**
Demographics				
Age at diagnosis, y	73 ± 4	73 ± 4	73 ± 4	0.77
Age at enrollment, years	77 ± 5	77 ± 4	77 ± 4	0.83
Female	44 (64)	31 (60)	13 (77)	0.21
Weight, Kg	64 ± 15	63 ± 14	68 ± 18	0.31
Height, cm	163 ± 9	162 ± 10	163 ± 6	0.86
BSA, m^2	1.70 ± 0.22	1.68 ± 0.22	1.74 ± 0.24	0.37
BMI, Kg/m^2	24 ± 5	24 ± 4	25 ± 6	0.28
Clinical and echocardiographic parameters				
WHO-FC I-II	32 (46)	23 (44)	9 (53)	0.44
Systemic hypertension	45 (65)	33 (64)	12 (71)	0.59
Diabetes	15 (22)	12 (23)	3 (18)	0.75
CAD	17 (25)	13 (25)	4 (24)	0.90
Permanent AF	3 (4)	2 (4)	1 (6)	0.72
Pulmonary disease	31 (45)	26 (50)	5 (29)	0.14
CKD	26 (38)	17 (33)	9 (53)	0.09
Previous/current cancer	8 (12)	6 (12)	2 (12)	0.98
SBP, mmHg	124 ± 15	123 ± 15	124 ± 13	0.83
DBP, mmHg	72 ± 9	72 ± 9	71 ± 9	0.64
SO_2, %	95 [93; 97]	95 [93; 97]	95 [94; 97]	0.75
6MWD, meters	304 ± 199	312 ± 112	274 ± 147	0.37
LVEF <50%	4 (6)	3 (6)	1 (6)	0.73
LVH	17 (25)	11 (21)	6 (35)	0.19
LA dilation	31 (45)	22 (42)	9 (53)	0.52
TAPSE, mm	20 ± 5	20 ± 5	20 ± 4	0.86
TRV, m/s	3.81 ± 0.76	3.93 ± 0.71	3.41 ± 0.80	**0.02**
TAPSE/TRV	5.6 ± 1.9	5.4 ± 1.8	6.4 ± 2.1	0.07
RVSP, mmHg	57 [42; 77]	62 [45; 83]	45 [36; 63]	**0.02**
RA dilation	53 (77)	40 (77)	13 (77)	0.91
Pericardial effusion	11 (16)	10 (19)	1 (6)	0.27
Most recent RHC				
RAP, mmHg	7 [4; 10]	6 [3; 10]	8 [7; 11]	**0.02**
mPAP, mmHg	41 ± 10	42 ± 11	37 ± 7	0.07
dPAP, mmHg	25 ± 9	26 ± 10	23 ± 6	0.20
sPAP, mmHg	70 ± 20	72 ± 21	62 ± 16	0.12
PAWP, mmHg	11 ± 4	10 ± 3	16 ± 2	**<0.001**
RAP/PAWP ratio	0.60 [0.46; 0.75]	0.63 [0.45; 0.76]	0.53 [0.46; 0.73]	0.72
PVR, WU	7.39 ± 4.53	8.40 ± 4.73	4.29 ± 1.53	**0.001**

Cardiac output, L/min	4.59 ± 1.43	4.41 ± 1.50	5.14 ± 1.07	0.07
Cardiac index, L/min/m^2	2.74 ± 0.81	2.62 ± 0.81	3.09 ± 0.75	**0.04**
Diagnosis to last RHC interval, months	15 [4; 33]	13 [2; 30]	18 [6; 52]	0.26
Treatment				
No PAH therapy	6 (9)	5 (10)	1 (6)	0.64
Bosentan	-	-	-	
Ambrisentan	17 (25)	13 (25)	4 (24)	0.90
Macitentan	32 (46)	24 (46)	8 (47)	0.79
ERA	49 (71)	37 (71)	12 (71)	0.96
Sildenafil	22 (32)	14 (27)	8 (47)	0.12
Tadalafil	25 (36)	19 (37)	6 (35)	0.93
Riociguat	3 (4)	3 (6)	0	0.57
PDE5i/GCs	50 (73)	36 (69)	14 (82)	0.29
Dual oral combination therapy	36 (52)	26 (50)	10 (59)	0.53
Selexipag	6 (9)	4 (8)	2 (12)	0.63
Treprostinil	-	-	-	
Epoprostenol i.v.	1 (1)	1 (2)	0	0.75
Inhaled iloprost	2 (3)	2 (4)	0	0.57
Beta blockers	9 (13)	7 (14)	2 (12)	0.86
RASi	23 (33)	12 (23)	11 (65)	**0.002**
MRA	32 (46)	26 (50)	6 (35)	0.35
Furosemide	57 (83)	44 (85)	13 (77)	0.44
Digoxin	6 (9)	5 (10)	1 (6)	0.64
Amiodarone	9 (13)	4 (8)	5 (29)	**0.04**
Warfarin	11 (16)	9 (17)	2 (12)	0.65
DOAC	11 (16)	7 (14)	4 (24)	0.27
SAPT	22 (32)	16 (31)	6 (35)	0.73
Statins	26 (38)	17 (33)	9 (53)	0.14
Ezetimibe	3 (4)	3 (6)	0	0.57
Antidiabetic drugs	12 (17)	10 (19)	2 (12)	0.72

Characteristics of patients with and without a left heart disease (LHD) phenotype according to the haemodynamic criteria definition (used in both main and secondary analysis). Data are expressed as n (%), mean ± SD or median [IQR], as appropriate.

LHD, left heart disease; *BSA,* body surface area; *BMI,* body mass index; *PAH,* pulmonary arterial hypertension; *WHO-FC,* World Health Organization functional class; *CAD,* coronary artery disease; *AF,* atrial fibrillation; *CKD,* chronic kidney disease; *SBP,* systolic blood pressure; *DBP,* diastolic blood pressure; *SO_2,* oxygen saturation; *6MWD,* six minute walking distance; *LVEF,* left ventricular ejection

fraction; *LVH,* left ventricular hypertrophy; *LA,* left atrium; *TAPSE,* tricuspid annular plane systolic excursion; *TRV,* tricuspid regurgitant velocity; *RVSP,* right ventricular systolic pressure; *RA,* right atrium; *RHC,* right heart catheterization; *RAP,* right atrial pressure; *mPAP, dPAP and sPAP* for mean, diastolic and systolic pulmonary artery pressure; *PAWP,* pulmonary artery wedge pressure; *PVR,* pulmonary vascular resistance; *ERA,* endothelin receptor antagonist; *PDE5i,* phosphodiesterase type 5 inhibitor; *GCs,* guanylate cyclase stimulator; *RASi,* renin-angiotensin system inhibitors; *MRA,* mineralcorticoid receptor antagonist; *DOAC,* direct oral anticoagulant; *SAPT,* single antiplatelet therapy.

Table S4. Baseline characteristics of the study population according to the secondary analysis.

	**Overall (N=69)**	**No LHD profile (N=32)**	**LHD phenotype (N=37)**	**P**
Demographics				
Age at diagnosis, y	73 ± 4	73 ± 4	73 ± 4	0.53
Age at enrolment, years	77 ± 5	76 ± 4	78 ± 3	0.12
Female	44 (64)	18 (56)	26 (70)	0.23
Weight, Kg	64 ± 15	62 ± 13	67 ± 16	0.16
Height, cm	163 ± 9	161 ± 9	164 ± 8	0.10
BSA, m^2	1.70 ± 0.22	1.65 ± 0.21	1.74 ± 0.23	0.14
BMI, Kg/m^2	24 ± 5	24 ± 4	25 ± 5	0.40
Clinical and echocardiographic parameters				
WHO-FC I-II	32 (46)	15 (47)	17 (46)	0.89
Systemic hypertension	45 (65)	17 (53)	28 (76)	0.05
Diabetes	15 (22)	2 (6)	13 (35)	**0.004**
CAD	17 (25)	6 (19)	11 (30)	0.29
Permanent AF	3 (4)	1 (3)	2 (5)	0.64
Pulmonary disease	31 (45)	17 (53)	14 (38)	0.20
CKD	26 (38)	5 (16)	21 (57)	**<0.001**
Previous/current cancer	8 (12)	5 (16)	3 (8)	0.33
SBP, mmHg	124 ± 15	125 ± 16	123 ± 14	0.68
DBP, mmHg	72 ± 9	74 ± 9	70 ± 9	0.11
SO_2, %	95 [93; 97]	94 [93; 96]	96 [94; 97]	0.31
6MWD, meters	304 ± 199	326 ± 131	279 ± 102	0.18
LVEF <50%	4 (6)	1 (3)	3 (8)	0.63
LVH	17 (25)	3 (9)	14 (38)	**0.005**
LA dilation	31 (45)	7 (22)	24 (65)	**0.001**
TAPSE, mm	20 ± 5	21 ± 5	20 ± 5	0.64
TRV, m/s	3.81 ± 0.76	3.87 ± 0.69	3.75 ± 0.82	0.53
TAPSE/TRV	5.6 ± 1.9	5.5 ± 1.9	5.7 ± 2.0	0.79
RVSP, mmHg	57 [42; 77]	65 [43; 77]	54 [40; 85]	0.50
RA dilation	53 (77)	24 (75)	29 (78)	0.29
Pericardial effusion	11 (16)	6 (19)	5 (14)	0.55
Most recent RHC				
RAP, mmHg	7 [4; 10]	5 [3; 8]	8 [5; 10]	**0.03**

mPAP, mmHg	41 ± 10	42 ± 12	40 ± 9	0.48
dPAP, mmHg	25 ± 9	27 ± 11	24 ± 7	0.16
sPAP, mmHg	70 ± 20	71 ± 21	68 ± 19	0.47
PAWP, mmHg	11 ± 4	10 ± 3	12 ± 5	0.64
RAP/PAWP ratio	0.60 [0.46; 0.75]	0.59 [0.39; 0.72]	0.64 [0.47; 0.81]	0.28
PVR, WU	7.39 ± 4.53	8.50 ± 5.26	6.42 ± 3.58	0.06
Cardiac output, L/min	4.59 ± 1.43	4.39 ± 1.66	4.77 ± 1.19	0.28
Cardiac index, L/min/m^2	2.74 ± 0.81	2.63 ± 0.93	2.83 ± 0.70	0.32
Diagnosis to last RHC interval, months	15 [4; 33]	12 [4; 30]	18 [4; 38]	0.48
Treatment				
No PAH therapy	6 (9)	2 (6)	4 (11)	0.50
Bosentan	-	-	-	
Ambrisentan	17 (25)	7 (22)	10 (27)	0.62
Macitentan	32 (46)	17 (53)	15 (41)	0.35
ERA	49 (71)	24 (75)	25 (68)	0.50
Sildenafil	22 (32)	9 (28)	13 (35)	0.53
Tadalafil	25 (36)	10 (31)	15 (41)	0.42
Riociguat	3 (4)	3 (9)	0	0.06
PDE5i/GCs	50 (73)	22 (69)	28 (76)	0.52
Dual oral combination therapy	36 (52)	16 (50)	20 (54)	0.74
Selexipag	6 (9)	3 (9)	3 (8)	0.85
Treprostinil	-	-	-	
Epoprostenol i.v.	1 (1)	1 (3)	0	0.29
Inhaled iloprost	2 (3)	1 (3)	1 (3)	0.92
Beta blockers	9 (13)	3 (9)	6 (16)	0.40
RASi	23 (33)	6 (19)	17 (46)	**0.02**
MRA	32 (46)	14 (44)	18 (49)	0.69
Furosemide	57 (83)	24 (75)	33 (89)	0.12
Digoxin	6 (9)	3 (9)	3 (8)	0.85
Amiodarone	9 (13)	4 (13)	5 (14)	0.87
Warfarin	11 (16)	5 (16)	6 (16)	0.91
DOAC	11 (16)	5 (16)	6 (16)	0.91
SAPT	22 (32)	7 (22)	15 (41)	0.10
Statins	26 (38)	11 (34)	15 (41)	0.60
Ezetimibe	3 (4)	0	3 (8)	0.10
Antidiabetic drugs	12 (17)	1 (3)	11 (30)	**0.004**

Characteristics of patients with and without a left heart disease (LHD) phenotype according to the secondary analysis criteria. Data are expressed as n (%), mean ± SD or median [IQR], as appropriate.

LHD, left heart disease; *BSA,* body surface area; *BMI,* body mass index; *PAH,* pulmonary arterial hypertension; *WHO-FC,* World Health Organization functional class; *CAD,* coronary artery disease; *AF,* atrial fibrillation; *CKD,* chronic kidney disease; *SBP,* systolic blood pressure; *DBP,* diastolic blood pressure; *SO_2,* oxygen saturation; *6MWD,* six minute walking distance; *LVEF*, left ventricular ejection fraction; *LVH,* left ventricular hypertrophy; *LA,* left atrium; *TAPSE,* tricuspid annular plane systolic excursion; *TRV,* tricuspid regurgitant velocity; *RVSP,* right ventricular systolic pressure; *RA,* right atrium; *RHC,* right heart catheterization; *RAP,* right atrial pressure; *mPAP, dPAP and sPAP* for mean, diastolic and systolic pulmonary artery pressure; *PAWP,* pulmonary artery wedge pressure; *PVR,* pulmonary vascular resistance; *ERA,* endothelin receptor antagonist; *PDE5i,* phosphodiesterase type 5 inhibitor; *GCs,* guanylate cyclase stimulator; *RASi,* renin-angiotensin system inhibitors; *MRA,* mineralcorticoid receptor antagonist; *DOAC,* direct oral anticoagulant; *SAPT,* single antiplatelet therapy.

Table S5. Baseline characteristics of the study population according to the secondary analysis clinical criteria suggestive for left heart disease.

	**Overall (N=69)**	**No LHD profile (N=40)**	**LHD phenotype (N=29)**	**P**
Demographics				
Age at diagnosis, y	73 ± 4	72 ± 4	74 ± 4	0.11
Age at enrolment, years	77 ± 5	76 ± 4	78 ± 3	**0.03**
Female	44 (64)	23 (58)	21 (73)	0.20
Weight, Kg	64 ± 15	63 ± 13	67 ± 18	0.21
Height, cm	163 ± 9	161 ± 9	165 ± 9	0.09
BSA, m^2	1.70 ± 0.22	1.67 ± 0.20	1.74 ± 0.25	0.19
BMI, Kg/m^2	24 ± 5	24 ± 4	25 ± 6	0.55
Clinical and echocardiographic parameters				
WHO-FC I-II	32 (46)	20 (50)	12 (42)	0.66
Systemic hypertension	45 (65)	23 (58)	22 (76)	0.11
Diabetes	15 (22)	4 (10)	11 (38)	**0.005**
CAD	17 (25)	10 (25)	7 (24)	0.94
Permanent AF	3 (4)	1 (3)	2 (7)	0.38
Pulmonary disease	31 (45)	18 (45)	13 (45)	0.99
CKD	26 (38)	11 (28)	15 (52)	0.05
Previous/current cancer	8 (12)	7 (18)	1 (3)	0.07
SBP, mmHg	124 ± 15	123 ± 16	124 ± 14	0.80
DBP, mmHg	72 ± 9	73 ± 9	70 ± 9	0.33
SO_2, %	95 [93; 97]	94 [93; 96]	97 [94; 97]	0.18
6MWD, meters	304 ± 199	315 ± 132	282 ± 89	0.38
LVEF <50%	4 (6)	1 (2)	3 (10)	0.36
LVH	17 (25)	3 (8)	14 (48)	**<0.001**
LA dilation	31 (45)	7 (18)	24 (83)	**<0.001**
TAPSE, mm	20 ± 5	20 ± 5	20 ± 4	0.80
TRV, m/s	3.81 ± 0.76	3.73 ± 0.76	3.92 ± 0.77	0.32
TAPSE/TRV	5.6 ± 1.9	5.8 ± 2.0	5.4 ± 1.9	0.42

RVSP, mmHg	57 [42; 77]	58 [40; 70]	57 [44; 89]	0.39
RA dilation	53 (77)	29 (73)	24 (83)	0.05
Pericardial effusion	11 (16)	7 (18)	4 (14)	0.68
Most recent RHC				
RAP, mmHg	7 [4; 10]	6 [3; 8]	8 [6; 10]	0.05
mPAP, mmHg	41 ± 10	41 ± 11	41 ± 9	0.87
dPAP, mmHg	25 ± 9	26 ± 10	25 ± 7	0.71
sPAP, mmHg	70 ± 20	69 ± 20	70 ± 20	0.91
PAWP, mmHg	11 ± 4	11 ± 4	11 ± 5	0.91
RAP/PAWP ratio	0.60 [0.46; 0.75]	0.55 [0.35; 0.72]	0.67 [0.53; 0.83]	**0.04**
PVR, WU	7.39 ± 4.53	7.66 ± 5.05	7.00 ± 3.75	0.56
Cardiac output, L/min	4.59 ± 1.43	4.55 ± 1.63	4.65 ± 1.12	0.77
Cardiac index, L/min/m^2	2.74 ± 0.81	2.74 ± 0.96	2.73 ± 0.58	0.97
Diagnosis to last RHC interval, months	15 [4; 33]	15 [5; 34]	15 [2; 32]	0.73
Treatment				
No PAH therapy	6 (9)	2 (5)	4 (14)	0.20
Bosentan	-	-	-	
Ambrisentan	17 (25)	9 (23)	8 (28)	0.63
Macitentan	32 (46)	21 (53)	11 (38)	0.19
ERA	49 (71)	30 (75)	19 (66)	0.39
Sildenafil	22 (32)	14 (35)	8 (28)	0.51
Tadalafil	25 (36)	13 (33)	12 (41)	0.45
Riociguat	3 (4)	3 (8)	0	0.13
PDE5i/GCs	50 (73)	30 (75)	20 (69)	0.58
Dual oral combination therapy	36 (52)	22 (55)	14 (48)	0.58
Selexipag	6 (9)	4 (10)	2 (7)	0.65
Treprostinil	-	-	-	
Epoprostenol i.v.	1 (1)	1 (3)	0	0.40
Inhaled iloprost	2 (3)	1 (3)	1 (3)	0.82
Beta blockers	9 (13)	3 (8)	6 (21)	0.11
RASi	23 (33)	13 (33)	10 (35)	0.86
MRA	32 (46)	17 (43)	15 (52)	0.42
Furosemide	57 (83)	29 (73)	28 (97)	**0.009**
Digoxin	6 (9)	3 (8)	3 (10)	0.68
Amiodarone	9 (13)	7 (18)	2 (7)	0.22
Warfarin	11 (16)	6 (15)	5 (17)	0.75
DOAC	11 (16)	7 (18)	4 (14)	0.72
SAPT	22 (32)	10 (25)	12 (41)	0.15

Statins	26 (38)	17 (43)	9 (31)	0.33
Ezetimibe	3 (4)	0	3 (10)	**0.04**
Antidiabetic drugs	12 (17)	3 (8)	9 (31)	**0.01**

Characteristics of patients with and without a left heart disease (LHD) phenotype according to the clinical criteria of the secondary analysis. Data are expressed as n (%), mean ± SD or median [IQR], as appropriate.

LHD, left heart disease; *BSA,* body surface area; *BMI,* body mass index; *PAH,* pulmonary arterial hypertension; *WHO-FC,* World Health Organization functional class; *CAD,* coronary artery disease; *AF,* atrial fibrillation; *CKD,* chronic kidney disease; *SBP,* systolic blood pressure; *DBP,* diastolic blood pressure; *SO_2,* oxygen saturation; *6MWD,* six minute walking distance; *LVEF*, left ventricular ejection fraction; *LVH*, left ventricular hypertrophy; *LA,* left atrium; *TAPSE,* tricuspid annular plane systolic excursion; *TRV,* tricuspid regurgitant velocity; *RVSP,* right ventricular systolic pressure; *RA,* right atrium; *RHC,* right heart catheterization; *RAP,* right atrial pressure; *mPAP, dPAP and sPAP* for mean, diastolic and systolic pulmonary artery pressure; *PAWP,* pulmonary artery wedge pressure; *PVR,* pulmonary vascular resistance; *ERA,* endothelin receptor antagonist; *PDE5i,* phosphodiesterase type 5 inhibitor; *GCs,* guanylate cyclase stimulator; *RASi,* renin-angiotensin system inhibitors; *MRA,* mineralcorticoid receptor antagonist; *DOAC,* direct oral anticoagulant; *SAPT,* single antiplatelet therapy.
